# Coiled central venous catheter in superior vena cava

**DOI:** 10.4103/0019-5049.68396

**Published:** 2010

**Authors:** Pramendra Agrawal, Babita Gupta, Nita D’souza

**Affiliations:** Department of Anaesthesiology, Jai Prakash Narayan Apex Trauma Centre, All India Institute of Medical Sciences, New Delhi, India

Sir,

Central venous pressure (CVP) monitoring is a simple, relatively inexpensive method of assessing a patient’s circulating blood volume, cardiac status and vasomotor tone. It is essential to be aware of the inherent fallacies and inadequacies of the information derived. Inaccurate measurements are often obtained by the aberrant lodgement of the central venous catheter (CVC) tip.

**Figure 1:**
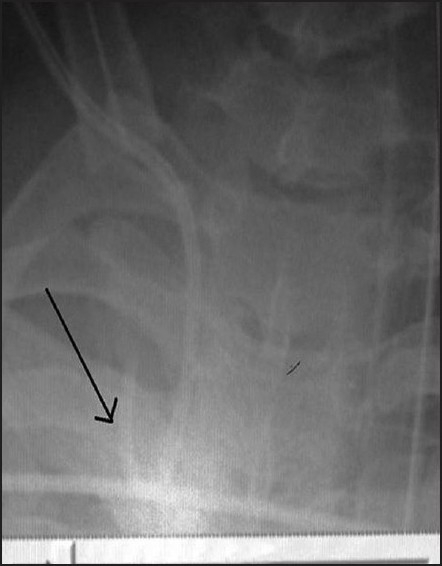
Coiled CVC in superior vena cava

In continuation of the previously published letter to editor concerning misdirected CVC, we describe an unusual case of CVC coiling in the superior vena cava (SVC) leading to falsely high CVP measurement. A 55 year old male patient was brought to the emergency room (ER) with head injury, blunt trauma abdomen with haemodynamic instability. He was further posted for an emergency laparotomy. In view of the clinical condition of the patient and the need to know intravascular volume status, a 7 French triple lumen CVC was inserted in the right internal jugular vein (IJV) in the operating room (OR). All the three ports were checked for free flow of blood and the CVC was fixed at 11cm at skin level. On connecting the transducer to the monitor, ideal waveform was absent. Intra operatively CVP tracing was suboptimal despite the change of transducer, the cable, flushing the unit and repeated zeroing. Post operatively the patient was shifted to intensive care unit on ventilator support for further management. Chest radiograph revealed coiled CVC in the SVC [Figure 1]. Hence it was removed and right subclavian vein was cannulated.

The correct placement of the CVC tip is an important factor in obtaining accurate CVP measurements. Malposition of a CVC may occur at the time of insertion or later as a result of spontaneous migration due to anatomic positioning or pressure changes within the thoracic cavity.[1] There has been case report on CVC folding back during guide-wire removal inside IJV.[2] In our case CVC coiling inside SVC was unusual as it is a large calibre vessel with high flows. There was no anatomical vascular abnormality and no manufacturing defect in CVC. The abutting of the guide wire against the wall of the SVC probably caused the coiling of CVC in the SVC. The J tip of guide wire probably was unknowingly directed cephalad while insertion, which could have caused CVC to further angulate in the upward direction over the guide wire.

In conclusion, inaccurate CVP measurements or inability to obtain an ideal wave from tracing are suggestive of an undesirable location of the catheter tip. Awareness of this possibility and careful review of the CVC tip position on X-ray pictures in suspicious cases are important. Inaccurate CVP readings lead to improper assessment of the intravascular status of the patient. Careful clinical co-relation under such circumstances is essential. Roentgenograms after insertion of CVC are essential to eliminate this problem, which is often encountered in clinical practice.
